# Enhanced forest fire evacuation planning using real-time sensor and GPS algorithm

**DOI:** 10.1038/s41598-024-71052-8

**Published:** 2024-08-29

**Authors:** Vishal Sharma, Deepali Nagpal, Suhasini Monga, Ahmad Almogren, Durgesh Srivastava, Ayman Altameem, Jaeyoung Choi

**Affiliations:** 1 Chitkara University Institute of Engineering and Technology, Chitkara University, Rajpura, Punjab India; 2 Department of Computer Science, College of Computer and Information Sciences, King Saud University, 11633 Riyadh, Saudi Arabia; 3 Department of Natural and Engineering Sciences, College of Applied Studies and Community Services, King Saud University, 11543 Riyadh, Saudi Arabia; 4 School of Computing, Gachon University, Seongnam-si, 13120 Republic of Korea

**Keywords:** Environmental social sciences, Natural hazards, Engineering

## Abstract

Forest fires are the source of countless fatalities and extreme economic repercussions. The safe evacuation of residents of an area affected by forest fires is the highest priority of local authorities, and finding the most optimal course of action has been a primary research focus for years. Previous studies over several decades have attempted to find an optimal solution using the applications of bug navigation systems, road network reconfiguration, graph traversals, swarm optimization, etc. The author, with the motivation to prevent human casualties at the time of such calamity, presents a novel study which solves the problem in nearly linear time computation, surpassing the performance standards of previous research, and accommodates the unpredictability of the spread of forest fires. This includes a proposal of an algorithm which builds upon the application of Spielman and Teng’s Electrical Circuit Approach to solve for maximum flow in a network and implements this with real-time sensor and Global Positioning System input.

## Introduction

**Fig. 1:**
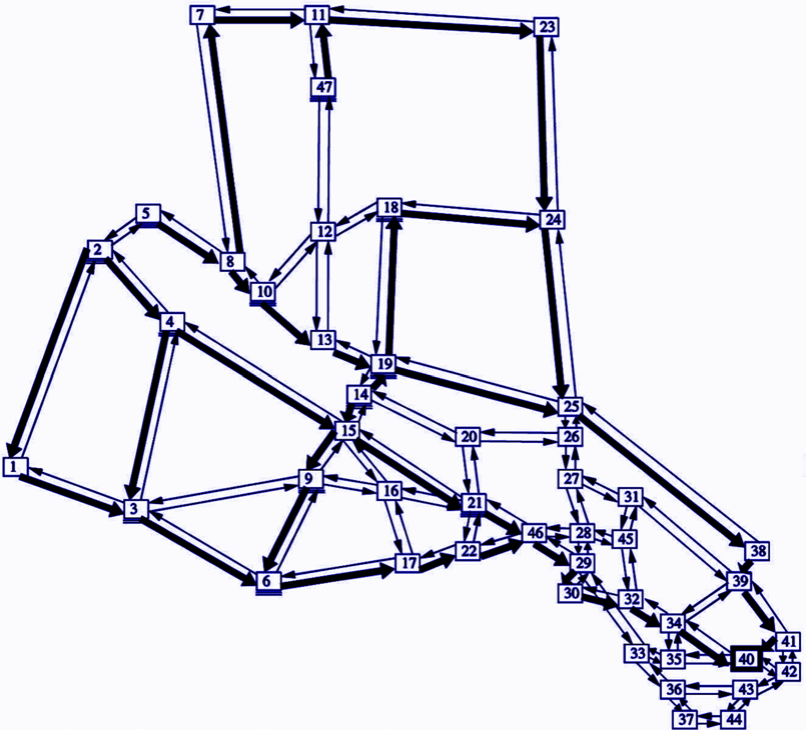
An example of an urban road network in a residential area^27^.

Forest fires are very difficult to control or even limit to a certain area, which makes populous areas vulnerable to its dangerous effects. The necessity at such a point is to ensure the safe evacuation of all residents of such areas which are or may come in the wildfire zone. In such scenarios, residents try to leave as the roads of the town/city are congested by multiple vehicles attempting to move towards one of the exits. This unplanned evacuation of residents without proper coordination results in innumerable loss of lives which could have been prevented. It is therefore the responsibility of local authorities to provide the residents with proper directions out of the town/city so as to ensure a well-ordered evacuation and consequently, the prevention of a large-scale disaster. Several studies have been conducted over finding the optimal paradigm of evacuation. Elementary research has focused on evacuee proximity clustering and static evacuation^1^ applied with the fundamentals of maximum flow^2^. Evacuation planning research is categorized into three major categories: empirical studies of pedestrian behavior, mathematical models for simulation, and models for determining optimal evacuation plans or design solutions^3^. More advanced literature has focused on the last two research challenges, including, graph traversal^4^, bug navigation systems^5^, road network reconfiguration^6,7^, data aggregation and genetic algorithms integrated with centralized Decision Support System (DDS)^8^. This paper proposes an algorithm addressing model as well as optimization solutions, which aims to solve the fire evacuation problem for residential areas, as depicted in Fig. 1 (S. Kim, 2008), building upon the existing ST-ECA study undertaken on network flow maximization using Laplacian matrix system calculations for electrical network flow, including the real-time fire sensor network inputs to compute the capacities of each road; and afterwards, using real-time GPS input from user’s device, which can be used to provide directions based on the updated road network output by the algorithm. The algorithm must present continuous iterations of each path in the shortest amount of time. It must ensure that the roads do not become congested and therefore, ensure a fast and ordered evacuation, with consideration to the roads or intersections that become inaccessible due to the spreading fire or high resident concentration.

Prominent work on bug navigation systems for evacuation was published by Haghpanah et al.^5^, which consisted of two algorithms: Dijkstra’s algorithm for selecting an evacuation route for each resident, and a collision avoidance algorithm which optimized the program by preventing two or more residents from being in the same physical space at one time. Even pedestrians were included in the algorithm’s computations. Jeon et al.^4^ conducted a study on finding the solution for this evacuation problem using road network reconfiguration using A* algorithm for pathfinding ^9,10^. With regards to the need for an alternative to Dijkstra’s algorithm in evacuation problems due to its inability to solve for the shortest route when cost is negative, a study was published by Kim et al.^6^, which uses A* algorithm for its shortest path guidance system. Neshat et al.^11^ used the same algorithm with an optimization for particle swarm path planning. Some prominent works include the paper by Goerigk et al.^8^ focusing on binary search algorithms with maximum flow algorithms and Tharwat et al.^12^ emphasizing the critical role of defining a suitable curve to achieve collision-free paths with attributes like shortest length and smoothness. Employing a model based on the Bézier curve fundamentals, the study introduces an advanced and optimized algorithm, presenting two variants with the focus on improving the generated Bézier curve model’s control points. Sheng et al.^13^ conducted research on human path planning making the use of Artificial Potential Field (APF) algorithm, emphasizing the practical application of their solution to overcome the difficulty of a goal unreachable surrounded by obstacles and the complexity of local minimum issues, the research suggests an enhanced repulsive force field function. Li et al.^14^ presents a new algorithm that merges an enhanced artificial fish swarm algorithm^14–16^ designed for path planning. This algorithm is coupled with continuously segmented Bézier curves, contributing to the seamless refinement of paths for mobile robots. Another subject gaining recent popularity has been the sparrow search algorithm^17,18^ particularly for the application of evacuation. It optimizes paths effectively but a desirable net optimal flow is not achieved^19,20^. X. Wei et al. conducted a study to modify the sparrow search algorithm by accommodating the Golden Sine Algorithm and nonlinear weight factor^17^. However, the study’s proposal primarily focuses on optimizing path finding and reducing path lengths but fails to be effective for a road network environment of evacuation and to deal with large randomness. More studies developed on swarm algorithms and generated solutions incorporating evolutionary algorithms^15,16^ have been proposed. Duchon et al.^21^ conducted research pertaining to path planning, employing a customized A* algorithm specifically designed for mobile robotic applications. However, it was found that the paths found using this algorithm tend to be a lot longer than the average. This would mean leaving a resident vulnerable for a longer period of time. Jha et al.^22^ presented a study which formulated the challenge of robot-motion planning framed within the context of a differential non-cooperative game. A drawback is that this is suited for machines, who in their ideal state of function, would not require online tracking of trajectory, hence rendering it unsuitable for real-life evacuation scenarios.

**Fig. 2:**
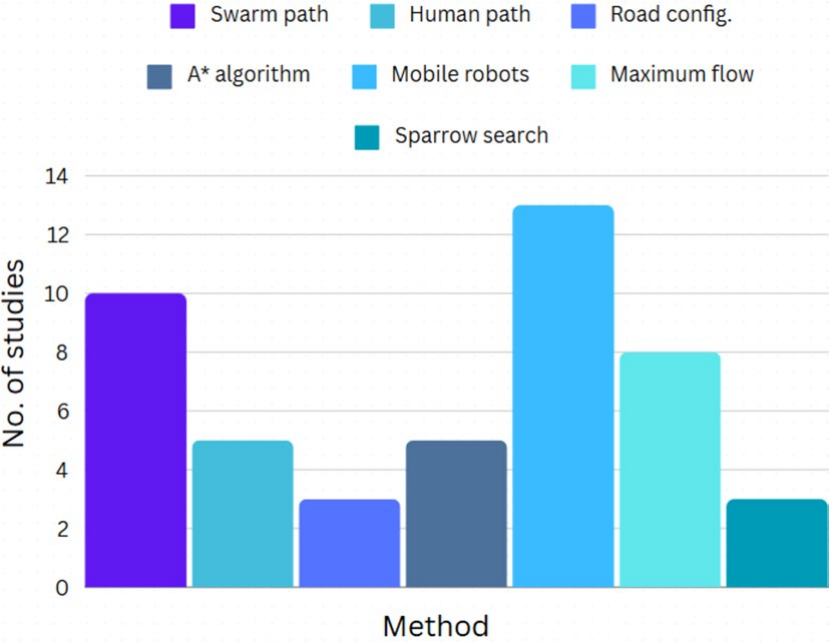
Studies done for finding optimal maximum flow over (2013–2023).

Figure 2 depicts methodology distribution of studies conducted for the years 2010–2024. Several studies have been conducted with their advantages and disadvantages^23^. The authors have made significant contributions to the subject. Hereafter, the results of the studies conducted on the viability of the above research is shared which helps in firstly, understanding as to how these models fail to solve the evacuation problem in its entirety and secondly, shapes the direction of the study conducted by the author to create and present the most optimal solution. The studies do not prove promising solutions based on this criterion. Table 1 summarizes the review of these papers.

**Table 1:** Merits and Demerits of the studies conducted on the problem.

Study	Author	Methodology	Merits	Demerits
Research on Evacuation Path Planning Based on Improved Sparrow Search Algorithm	Wei et al. (2024)	Implements an improved sparrow search algorithm with Golden Sine algorithm	Good solving abilities along with path optimization	Study focuses on path optimization while neglecting multi-objective optimization
Guide to evacuation based on A* algorithm for the shortest route search in case of fire system	Jeon et al. (2021)	Shortest route computed using A* algorithm	Better algorithm used than Dijkstra’s for path computations in the case of large-scale map	Time complexity of A* algorithm increases exponentially in the worst case. The unpredictability of fire’s spread is not taken into account
Application of bug navigation algorithms for large-scale agent-based evacuation modeling to support decision making	Haghpanah et al. (2021)	Bug navigation system used for agent-based evacuation	Uses the application of bug navigation to provide an optimized solution to provide decision making models	Unnecessary computations arise due to the use of bug navigation systems with regards to obstacle avoidance
RnR-SMART: Resilient smart city evacuation plan based on road network reconfiguration in outbreak response	Kim et al. (2021)	The model creates an evacuation plan for large-scale road network using RnR applications	Prioritizes low disaster risk over minimum time cost evacuation	Uses A* algorithm for path computations and hence, results in exponential time complexity in the worst case
A Comprehensive Evacuation Planning Model and Genetic Solution Algorithm	Goerigk et al. (2014)	Metaheuristic solution based upon genetic algorithms, with respect to data aggregation	Evaluates using genetic algorithm’s dependance on the degree of data aggregation	Does not address the fundamental drawbacks of presumption of deterministic environment
Intelligent Bezier curve-based path planning model using Chaotic Particle Swarm Optimization algorithm	Tharwat et al. (2019)	An optimized algorithm has been proposed presenting two variants to improve the generated control points	Addresses the optimization of the generate Bézier’s control points	Proposed model has only been studied in a static environment, but in an evacuation scenario, there can be several moving obstacles
An Improved Artificial Potential Field Algorithm for Virtual Human Path Planning	Sheng et al. (2010)	Enhances the repulsive force field function to mitigate issues associated with the unreachable goal and local minima problem	Adjusts the target point to serve as the global minimum within the artificial potential field algorithm	Fails to resolve the problem regarding local shock, leading to an unsmooth path
Path planning and smoothing of mobile robot based on improved artificial fish swarm algorithm	Li et al. (2022)	Algorithm fundamentals based upon the improved artificial fish swarm algorithm for path planning	It uses dynamic feedback horizons to mitigate convergence issues, incorporating Bessel curve theory	The solution was not tested in a real environment
Path planning with modified A star algorithm for a mobile robot	Ducho et al. (2014)	Finding paths by applying Jump Point Search (JPS) directly on A* algorithm for mobile robots	Minimum computational time as compared to other A* applications	The paths were found to be a lot longer than the average, which is not useful in an evacuation scenario
Game Theoretic Controller Synthesis for Multi-Robot Motion Planning-Part II: Policy-based Algorithms	Jha et al. (2015)	Leveraging Rapidly exploring Random Graphs (RRG) to propose the Feedback iNash-policy	Robot-motion planning framed within the context of a differential non-cooperative game	Not suitable for a real-time intensive data aggregation process of an evacuation

## Research gaps

The computational time required in maximum network flow algorithms is drastically lower than that of the studies discussed above (See Table 2), which becomes a crucial aspect given the time-sensitive nature of the problem. After decades of research, the complexity of algorithms to find a network’s maximum possible flow has been brought down drastically from Ford-Fulkerson’s O(n^2) complexity^24^. The algorithm went through several iterations^25,26^ and was improved by Spielman and Teng^21^, who used the approach of extending the Laplacian system of matrices in electrical circuits to compute the network’s maximum possible flow, bringing down the complexity to *O(n*^*1.33*^*)*^22^. Table 2 maps algorithms with their time complexities.

**Table 2:** Algorithms and methodologies and their average time complexities (Dimensions of graph: m x n).

Name	Theory	Time complexity
Swarm path finding	Employs decentralized agents that communicate and adapt locally to discover optimal paths	$\in \{ {\text{O}}({\text{n}}^{3} ) - {\text{O}}({\text{n}}^{2} )\}$
Human path planning	Cognitive decision-making and environmental awareness to navigate through spaces, simulating human-like movement	O(m + nlogn)
A* algorithm for networks	A* algorithm utilizes a heuristic-guided search to efficiently find the shortest path in a graph or grid	O(mnlog(mn))
Mobile Robots with Dijkstra’s	Calculates the shortest path in a graph by iteratively selecting the node with the lowest accumulated cost from the source	O(m + nlogn)
Ford-Fulkerson maximum flow	Max-flow algorithm that optimally determines the maximum flow in a network by iteratively augmenting paths	O(mn^2^)
ST-ECA	Computes max flow by imagining the network as an electric circuit	O(n^1.33^)

**Fig. 3:**
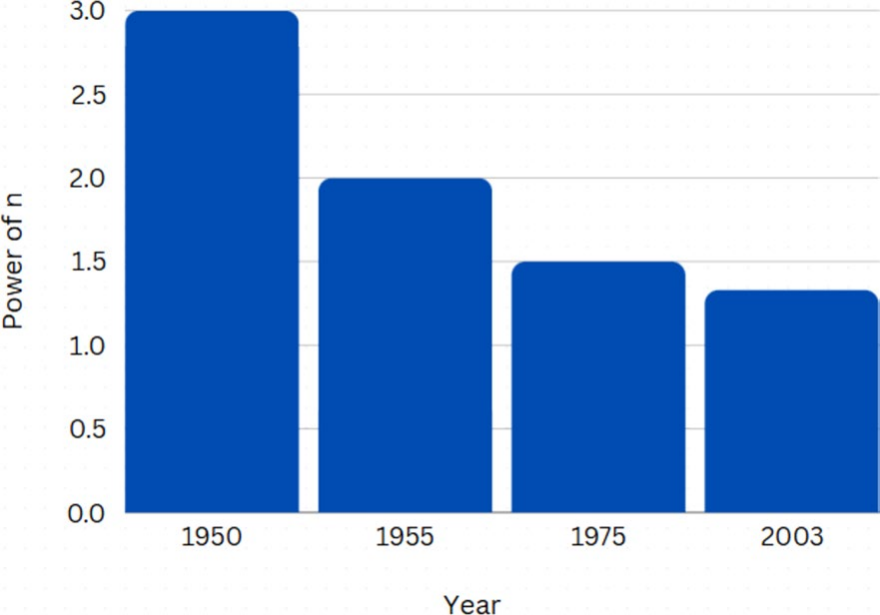
Time complexity of maximum flow algorithms the years, as a factor of the power of n.

ST-ECA imagined the network as an electrical circuit with a positive voltage at the source and sink as the ground and extended Laplacian matrices to find a general solution for the maximum network flow problem which consequently, results in an extremely optimized solution^21^. Figure 3 charts the time complexity of the maximum network flow algorithms over the years^28^.

As the fire radius increases, which has been found to be at an alarming rate, many roads/paths might become inaccessible. Research over the years has shown that Wireless Sensor Networks (WSN) provide the most efficient solution for forest fire detection^27,29^. This implies that the algorithm will have to run multiple iterations in real-time, assessing the situation of the fire and accordingly changing the output maximum flow with respect to the whole network. Maintaining a minimum computational stack is important for this element, which the above studies fail to incorporate either wholly or without computational overhead. Unpredictability of forest fire and the nature of its non-deterministic environment can be handled, as discussed in the following sections, by an ST-ECA based solution, which along with its most efficient computational time in the worst case (See Table 2), makes it the choice for the foundation of this study’s proposal. Obstacle avoidance is abstracted within road avoidance in the following proposal, preventing the unnecessary computations associated with it.

The author here after, has presented the study of employing these applications in regards to the proposal of an optimized algorithm for safe evacuation of residents of an area at the time of forest fires^30^.

## Methods

In preparation for the proposal of the solution, we present the problem definition for the scope of requirements of the application with respect to the merits and demerits of all the previous research conducted. Table 3 defines the problem statement with constraints and objects.

**Table 3:** Algorithms and methodologies and their average time complexities (Dimensions of graph: m x n).

Given	A road transportation network including Source nodes/vertices, exit vertices, all other intermediate vertices Set of edges Target flow value Capacities of edges, Edge weights
Output	Evacuation plan object with Flow *f* Set of forbidden edges
Object	Evacuation plan object with A flow *f* > target flow Minimize computation complexity Minimize total evacuation time
Constraint	Each edge has a capacity constraint along with flow conservation constraints

### Fundamentals

The fundamentals of ST-ECA state that the maximum flow in an undirected, capacitated graph is determined by solving a sequence of electrical flow problems. Each flow's value is obtained by resolving a set of linear differential equations contained in a Laplacian matrix. This method uses the Laplacian system, which allows the flow to be calculated in almost linear time.

### Spielman and Teng’s study

Every edge in the input graph is represented as a resistor with one unit of resistance in the foundations of the ST-ECA. The algorithm determines the electrical movement that results from current flowing from the point of origin to the sinks using this representation. Each edge's resistance is increased in accordance to the current passing through it in order to maintain the edge capacities in the computations. This augmentation serves to penalize edges that exceed their capacities, enabling the algorithm to compute the electrical flow using the adjusted resistances.

A source (s) and a sink (t) are the two defined vertices of a graph G (V, E), defined in Eq. (1).

1 $$G = \left( {E,V} \right)$$

A ratio U is defined and a capacity u_e_ ∈ Z^+^ is allocated to each edge e.

2 $$u_{e} \in Z +$$

3 $$U : = {\text{max}}_{e} u_{e}/ {\text{min}}_{e} u$$

The definition of the capacity, denoted as u(S), for a cut involves summing the capacities of the edges that have two endpoints, one in the set S and the other in the complement set V\S.

4 $$u\left( S \right): = \Sigma_{e \in E} u_{e} , \quad where\quad E\left( S \right) \subseteq E$$

Each edge is assigned a certain capacity *u*. Furthermore, a certain *resistance, r* > 0 is assigned to each edge. The congestion of an edge e for a given source-sink flow f is defined as the ratio of::

5 $$cong\,\,f\left( e \right) = \left| f \right|/u.$$

ST-ECAfurther defined a resistance r for each of the edges. After which a net energy ε_r_ is calculated for the entire network. The resistances are then stored in a vector R

6 $$r_{e} > 0,\quad where\quad e \in E, \, r \in R$$

7 $$\varepsilon_{r} \left( f \right): = \Sigma_{e} r_{e} f^{2} \left( e \right)$$

Now, the paper defined the application of the Laplacian system of matrices. It introduces the *Laplacian* matrix *L* of *G*, which is an n x n matrix. *L* is defined as follows:

8 $$L_{u,v} = \left\{ {\begin{array}{*{20}l} {\sum_{e \in E + \left( u \right) \cup E - \left( u \right)} c_{e} } \hfill & {if\,u = v,} \hfill \\ { - c_{e} } \hfill & {if\,e = \left( {u,v} \right)\,is\,an\,edge\,of\,G,\,and} \hfill \\ 0 \hfill & {otherwise} \hfill \\ \end{array} } \right.$$

The matrix R is expressed as the inverse of matrix C: R = C^−1^ resulting in a diagonal matrix with elements R_e,e_ = r_e._ Furthermore, matrix C is defined as a diagonal matrix of size m × m with diagonal elements C _e,e_ = c_e_ = 1/re.

9 $$\varepsilon_{r} \left( f \right): = \Sigma_{e} r_{e} f\left( e \right)^{2} = f^{T} Rf = \, \left| {\left| {R^{1/2} f} \right|} \right|^{2}$$

In addition to this, the study defines an incidence matrix B for edge-vertex pairings. A value of 1 is given for fixed source coordinates, -1 for sink coordinate and 0 for all other pairings.

Since, *f* is an electrical flow, its potential can be defined, such that for a vector *Φ ∈ R*^*v*^,

10 $$f\left( {u,v} \right) = \Phi_{v} - \Phi_{u} /r_{u,v}$$

That is,

11 $$f = CB^{T} \Phi = R^{ - 1} B^{T} \Phi$$

Applying *Bf* = *X*_*s,t*_, the equation *Bf* = *BCB*^*T*^*Φ* = *X*_*s,t*_ is derived, leading to the subsequent outcome:

12 $$\Phi = L^{t} X_{s,t}$$

This is the Moore–Penrose pseudo-inverse of L, denoted by Lt. As such, the following formula for the electrical flow f is found:

13 $$f = CB^{T} L^{t} X_{s,t}$$

This can be rewritten as:

14 $$\varepsilon_{r} \left( f \right): = \Phi^{T} L\Phi = f^{T} Rf \, = \, X_{s,t}^{T} L^{t} X_{s,t} ,\,{\text{where}}\,\Phi = L^{t} X_{s,t} ,L = BCB^{T}$$

**Fig. 4:**
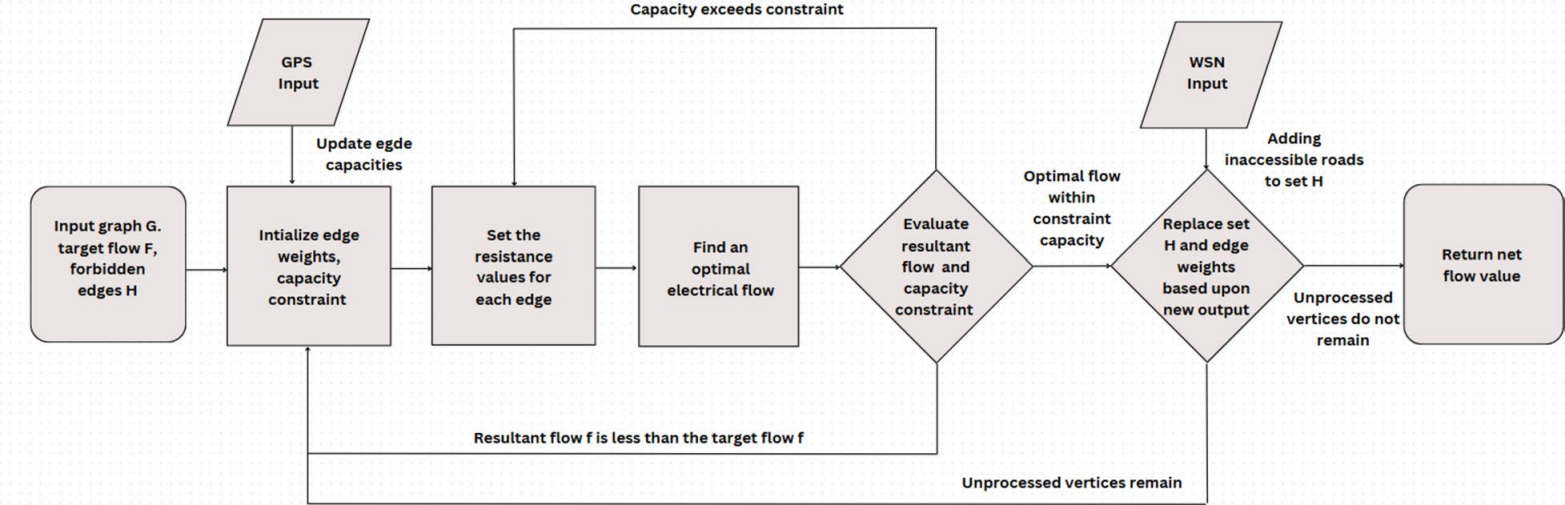
Flowchart depicting the working of the algorithm.

The algorithm is designed to minimize this energy, a task efficiently addressed through Laplacian principles. The algorithm uses this value in order to update the capacities of edges. Additionally, ST-ECA’s algorithm involves a set H consisting of forbidden edges of the network. Edges with congestion value exceeding a certain threshold value *p,* are added into the set of forbidden edges before the algorithm runs again in order to get the maximum possible flow. This exclusion is further extended to handle the roads affected by the spreading fires for evacuation applications. Figure 4 depicts the flow of this algorithm:

### Real world input and final net flow: WSN and GPS

Now, for the purpose of applying this algorithm in the real-life situation of wildfire evacuation, an efficient network for the area’s real-time monitoring is required. The solution is extended to include real-time inputs from GPS, used to dynamically update the capacities of each road/edge, and WSN, to provide real-time data on the status of roads and fire spread. The road (or an edge e) found to be compromised due to fire is added into the set of forbidden edges, *H*, as defined below*.*

15 $$H = HU\{ e \in E\}$$

GPS input computes resident clusters and updates the capacity of each edge in real-time. Let *u*^*o*^_*e*_ and *u*_*e*_*(t)* be edge capacities at time 0 and t, then,

16 $$u_{e} \left( t \right) = u^{o}_{e} + GPS\,input\,for\,edge\,e\,at\,time\,t$$

Thereafter, the resistance of an edge becomes:

17 $$r_{e} \left( t \right) = \max \left( {0, \, u_{e} \left( t \right) - f_{e} } \right)$$

Continuing the previous formulations, an algorithm which runs continuously through the evacuation efforts and computes net flow at each iteration can be expressed using the following.

18 $$F\left( t \right) \, = \frac{{\left( {1 - \in } \right)^{2} }}{{\left( {1 + \in } \right)N}} \left( {\mathop \sum \limits_{i = 1}^{N} f\left( t \right)^{i} } \right)$$

The algorithm’s net flow value can be used to dynamically adjust evacuation routes and to guide residents. Furthermore, this can be used to optimize relief and road clearance operations resulting in an overall efficient evacuation and prevention of casualties.

## Algorithm

*Initial: source:* center of the town, *sink:* submap’s exit for civilians. from the town center to the sink, the method computes a primary path, which is an augmented path from source to sink, a portion of the road network.

*Evacuation:* Each submap’s roads are subjected to the algorithm after receiving updates from GPS and WSN inputs. The resultant flow is used to update the capacities of passengers on each road.

*Proceed:* The intersections which are already computed as *sources* are added into the set of computed nodes. These intersections are eliminated from further computations of the algorithm. The roads deemed inaccessible by the central fire command are added into set H. Below (Table 4) is the pseudo code for the algorithm:

**Table 4:**
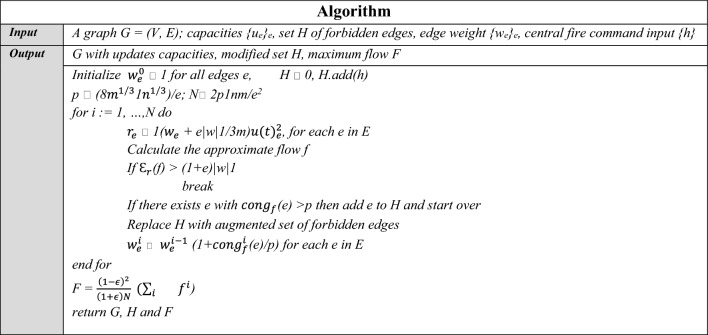
The Pseudo Code of the ST-ECA Algorithm.

**Fig. 5:**
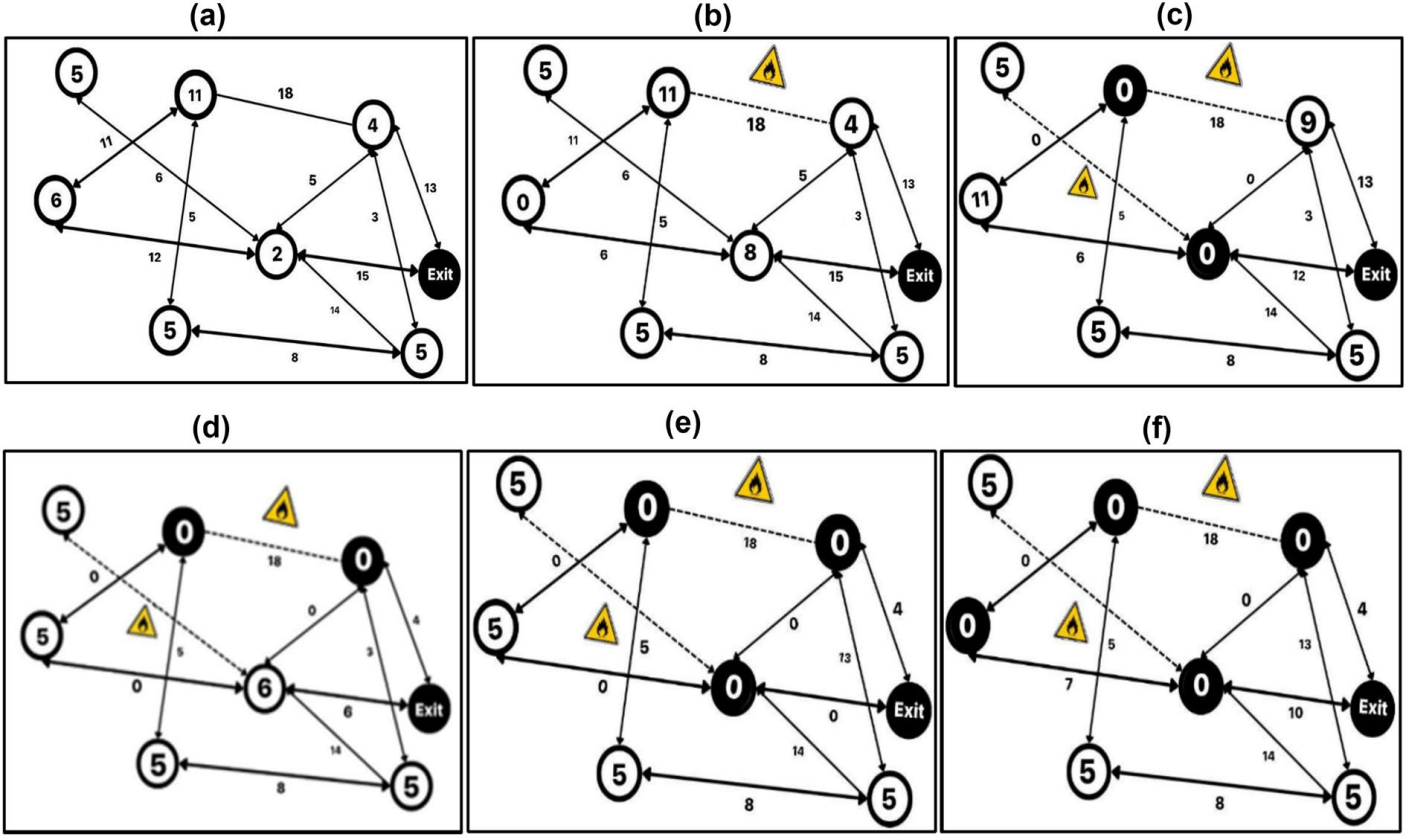
(**a**) Simulation Iteration, (**b**) Iteration-1, (**c**) Iteration-2, (**d**) Iteration-3, (**e**) Iteration-4, (**f**) Iteration-5.

Subsequent to the algorithm formation, an evaluation was conducted in a simulated environment. Based on the output of algorithm 2, the program application takes in the road network with updated capacities and can direct the individuals according to the flow value determined through real-time notifications. Each iteration of the algorithm results in an evacuation plan for the remaining evacuees, after updating capacities and edges using WSN and GPS input. A single thread computation is considered for the simulation according to which the starting time has been mentioned. Table 5 given below represents the data of the simulation conducted, and Fig. 5(a)–(f) depict the evacuation process.

**Table 5:** Evacuation simulation.

Start Time	Evacuation Plan	Blocked roads due to fire
0	V(1)–V(3)–V(4)–V(5)–V(8)–Exit	–
0	V(4)–V(5)–V(8)–V(7)–V(9)–V(10)–V(7)–Exit	–
1	V(4)–V(3)–V(1)–V(3)–V(0)–V(8)–V(7)–Exit	–
1	V(1)–V(3)–V(5)–V(6)–V(7)–V(8)–V(10)–Exit	–
2	V(4)–V(3)–V(1)–V(3)–V(0)–V(8)–V(7)–Exit	–
4	V(2)–V(5)–V(1)–V(7)–Exit	–
4	V1(11)–V(4)–V(5)–V(2) –V(6)–Exit	–
6	V(11)–V(5)–V(5)–V(8)–V(4)–Exit	–
7	V(11)–V(0)–V(9)–Exit	18
10	V(6)–Exit	18,6
11	V(5)–V(5)–V(0) Exit	18,6
12	V(5)–V(5)–V(0)–Exit	18,6
13	V(4)–V(7)–V(5)–V(2)–V(0)–V(8)–Exit	18,6

## Experimental data & calibration

This section describes the process of generating a random graph for our experimental setup. The generated graph serves as the basis for evaluating our algorithm. The graph can be represented by four main components, vertices (*V*), edges (*E*), edge capacities (*capacities: {u*_*e*_*}*_*e*_) and maximum capacity ${C}_{m}$. The list of indices from 0 to n − 1, where n is the number of vertices that are available, is used to establish the vertex set V. This guarantees that every vertex in the graph has a unique identity. This value is calibrated based on regular sizes of graphs and vertex count found in the relevant literature listed in this paper.

19 $$V = \left[ {0,1,2 \ldots n - 1} \right]$$

A sample of m unique edges—where m is the required edge count—is taken from the whole graph to produce the edge set (E). All potential edges between unique vertices define the whole graph E represents the resultant edge set. The edge count *m* is calibrated by examining regular networks such as Barabási-Albert and 55-node Swain. The edge set is defined below:

20 $$E \subseteq \{ \left( {i, \, j} \right) \, | \, i, \, j \in V, \, i$$

A capacity value is randomly allocated to each edge in the graph. The capacities are selected at random from an evenly distributed range that spans from 1 to *C*_*m*_ that has been defined. The list of capacities that is produced is referred to as capacities. *C*_*m*_ is calibrated by analyzing empirical data in the relevant literature listed in this study. The distribution *U(1,C*_*m​*_*)* ensures capacities fall within realistic bounds. The capacity set is mentioned below:

21 $$capacities = \left[ { c_{1} ,c_{2} , \ldots c_{n} } \right]\,where\quad c_{i} \sim U\left( {1,C_{m} } \right)$$

## Results and discussion

**Fig. 6:**
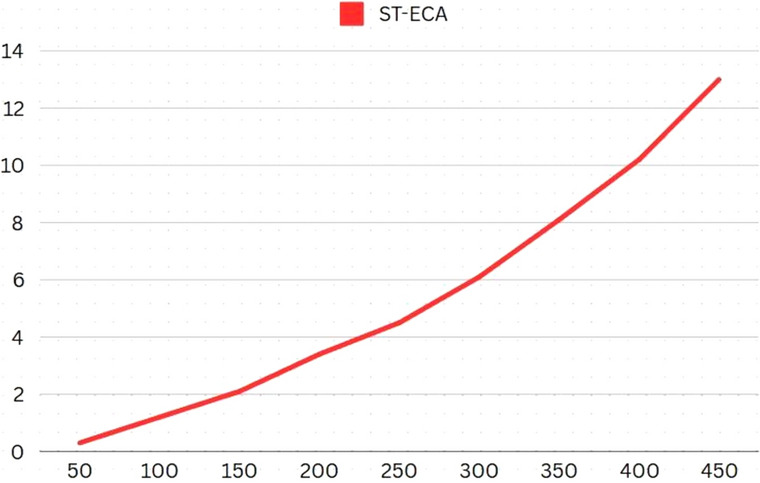
Results: Experiment Data Set-1.

To understand the effect on computational time with a linear increase in input, we have used the following dataset (Table 6). The vertical axis represents the values of aggregate flow of all edges: The results (Fig. 6) demonstrate that the algorithm’s worst case complexity is near-linear.

**Table 6:** Experiment Data Set-1.

V	50	100	150	200	250	300	350	400	450
E	80	130	180	230	280	330	380	430	480
C_m_	100	100	100	100	100	100	100	100	100

**Fig. 7:**
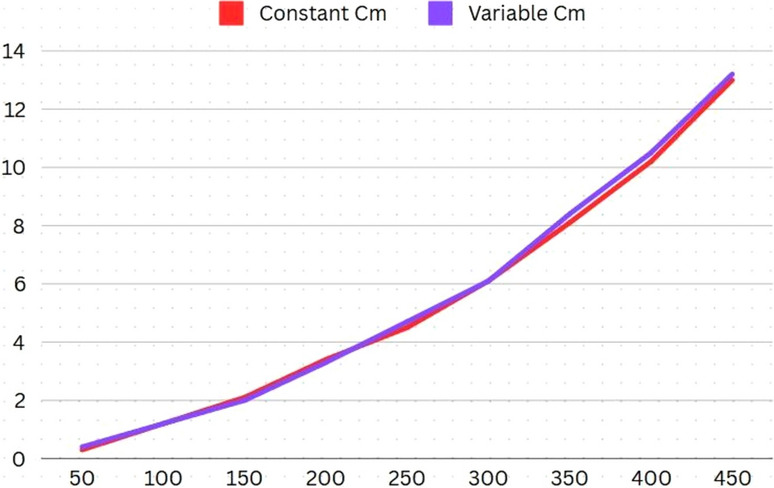
Results: Experiment Data Set-2.

To understand the change in computational time with respect to the linear increase of *C*_*m*_, the following dataset (Table 7) was used for experimentation. The results (Fig. 7) demonstrate that the linear rate of increase in *C*_*m*_ does not affect the computational time*.* This is a significant feature, as this means the resident count of a city would not affect the algorithm’s performance during the evacuation efforts.

**Table 7:** Experiment Data Set-2.

V	100	100	100	100	100	100	100	100	100
E	180	180	180	180	180	180	180	180	180
C_m_	100	200	300	400	500	600	700	800	900

**Fig. 8:**
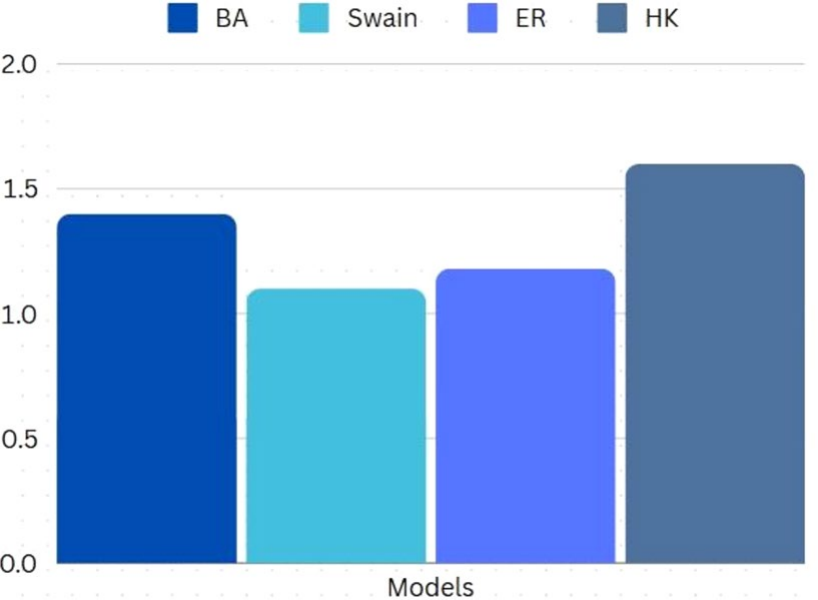
Results: Experiment Data Set-3.

Furthermore, it was necessary to study the algorithm’s performance for different network models. The following models were used for this experimentation, with *C*_*m*_ set to a randomly determined value. Below (Table 8) is the dataset. The results (Fig. 8) demonstrate close performance parity across multiple models showcasing the ability of the proposed solution to handle varied complexities of networks.

**Table 8:** Experiment Data Set-3.

Network model	Barabási-Albert(BA)	Swain	Erdős-Rényi (ER)	Holme-Kim(HK)
V	55	55	55	55
E	162	126	149	162

**Fig. 9:**
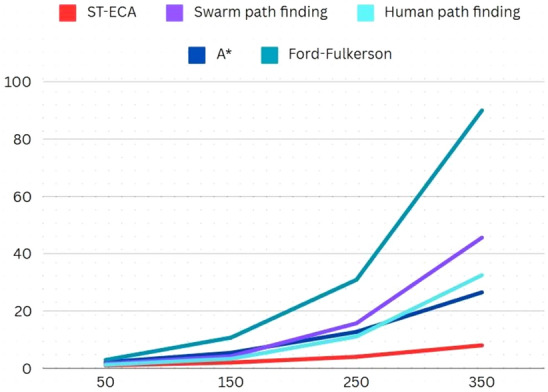
Results: Experiment Data Set-4.

In order to understand the algorithm’s performance with previous studies’ solutions, the following dataset was considered (Table 9). The number of vertices in each case were equal to half of the edge count, while *C*_*m*_ was a randomly determined value. The results (Fig. 9) indicate exceptional performance of this study’s proposed solution and the difference, as compared to other studies’ solutions, is greatly distinguishable at even low edge count inputs.

**Table 9:** Experiment Data Set-4.

Algorithms	Swarm path finding	Human path planning	A*	Ford-Fulkerson
E1	50	50	50	50
E2	150	150	150	150
E3	250	250	250	250
E4	350	350	350	350

### Limitations

The algorithm’s testing has not taken into account the GPS and WSN inputs’ latency. If a stack is not built for these data inputs then every iteration of the algorithm would have to wait for the next batch of updates. Additionally, the study has not been tested with a real city model.

## Conclusion

This paper proposes a comprehensive algorithm solution for resolving evacuation of residents of an area at the time of extreme forest fires. The solution assimilates WSN inputs and real-time GPS data of residents in order to accommodate the randomness of fire spread and the dynamic nature of resident movement and hence, safely evacuate them at the time of wildfire calamities. The author worked to expand upon ST-ECA on finding maximum network flow using Laplacian matrix applications. With the inclusion of multiple *source* and *sink* computations, real-time fire assessment and location data, the new algorithm has been presented as a holistic solution for an efficient evacuation operation. The subsequent studies would include the testing of the system in a simulation including all the defined variables under consideration, with their values changing as per their expected behavior in real-life. Furthermore, the study would be complete when the algorithm is deployed in a residential area at the time of forest fires.

## Data Availability

The datasets utilized in this research, along with the corresponding results, have been included within the paper. These datasets were generated exclusively for the purpose of conducting experiments to validate the proposed algorithm. These data were not acquired from external sources but were specifically crafted to suit the experimental requirements of the study. By providing access to the datasets alongside the results, we aim to facilitate transparency, reproducibility, and further analysis by interested readers.
